# Behavioral observations, heart rate and heart rate variability in horses following oral administration of a cannabidiol containing paste in three escalating doses (part 1/2)

**DOI:** 10.3389/fvets.2023.1305868

**Published:** 2023-12-11

**Authors:** Fabienne Eichler, Anna Ehrle, Katharina Charlotte Jensen, Natalie Baudisch, Hannah Petersen, Wolfgang Bäumer, Christoph Lischer, Mechthild Wiegard

**Affiliations:** ^1^Equine Clinic, Veterinary Hospital Freie Universität Berlin, School of Veterinary Medicine, Freie Universität Berlin, Berlin, Germany; ^2^Institute of Veterinary Epidemiology and Biostatistics, School of Veterinary Medicine, Freie Universität Berlin, Berlin, Germany; ^3^Institute of Pharmacology and Toxicology, School of Veterinary Medicine, Freie Universität Berlin, Berlin, Germany; ^4^Institute of Animal Welfare, Animal Behavior and Laboratory Animal Science, School of Veterinary Medicine, Freie Universität Berlin, Berlin, Germany

**Keywords:** behavior, CBD, equine, FaceSed, Horse Grimace Scale, sedation score

## Abstract

Cannabidiol (CBD) products have been proposed to exert stress- and anxiety-relieving effects in animals. Despite the increasing popularity of CBD for veterinary use, the available research detailing the effects of CBD in horses is limited. The aim of this study (part 1 of 2) was to analyze stress parameters via behavioral observations and heart rate monitoring in healthy horses following single oral administration of a CBD containing paste in different doses. Study products were two pastes for oral administration, one containing CBD and one containing no active ingredient. Pastes were applied as single administrations in consecutive trials with escalating dosages (doses: 0.2, 1.0, 3.0 mg CBD/kg) to a treatment (trial 1: *n* = 3, trial 2: *n* = 3, trial 3: *n* = 5 horses) and a control group (trial 1: *n* = 3, trial 2: *n* = 3, trial 3: *n* = 6 horses) with minimum wash-out periods of seven days in between. Behavioral parameters were evaluated using video recordings to score the levels of sedation including the horses' reactions to acoustic and visual stimuli. Facial expression was assessed using photographs. Evaluation was based on the previously described facial sedation scale for horses (FaceSed) and the Horse Grimace Scale. For baseline values, identical observations were recorded on the day before each paste administration. Both paste administration and behavioral evaluation were performed double blinded. Cardiac beat-to-beat (R-R) intervals were continuously recorded throughout the trial and assessed using heart rate and heart rate variability parameters. Statistical analysis included comparison between treatment and control group over escalating doses and time points using linear mixed models. The CBD paste was well tolerated, and no side effects were observed. Analysis of sedation scores and facial expressions did not indicate significant differences between treatment and control group over the escalating doses. The heart rate was neither reduced, nor were significant changes in heart rate variability observed compared to the control group. Main limitation of this study is the small sample size. Further research is required to determine adequate doses and indications for the use of CBD products in horses.

## 1 Introduction

Cannabidiol (CBD) belongs to the most well-known compounds of *Cannabis* plants and is gaining increasing attention in the field of veterinary medicine. Unlike Δ^9^-tetrahydrocannabinol (THC), CBD does not exhibit psychoactive properties (1, 2) but has been tested for analgesic, anti-inflammatory and anti-convulsant effects in companion animals (3–8). Additionally, the impact of CBD on anxiety and stress relief is currently under investigation. In humans, stress and anxiety are the most common indications for CBD use (9).

Mechanisms of action include various pathways: CBD may act as a ligand on serotonin_1A_ (5-HT_1A_) receptors (10–14) and inhibits the deactivation of endogenous cannabinoids such as anandamide (AEA) (15–17). AEA is a ligand of the endocannabinoid (eCB) system which regulates emotional responses and can reduce anxiety (12, 18, 19). CBD may also influence cannabinoid type 1 (CB_1_) receptors of the eCB system as an indirect agonist by increasing membrane fluidity and therefore modulating the constitutional activity of CB_1_ (12, 20, 21).

In humans and rodents, CBD has been reported to decrease heart rate and to show anxiolytic effects (9, 22–25). However, results remain inconsistent, as other studies could not confirm these findings to the same extent (26–29). Further effects of CBD include sedation, which has been reported in humans (30, 31). In dogs, surveys among US veterinarians and pet owners have reported that sedation is a perceived side effect following CBD or hemp supplementation (32–34). It was additionally suggested that CBD supplementation may decrease stress-related aggressive behavior (1). Another study could not identify significant alteration in daily activity or quality of sleep in dogs (35). There are few reports detailing the effect of CBD on equine behavior: One study found a reduction of reactivity without any significant effect on the heart rate (36). Other reports showed no effect of CBD on ataxia, sedation scores or overall equine behavior (37, 38). Two case reports described CBD as an effective treatment for stereotypic behavior such as crib-biting and mechanical allodynia (39, 40). The effect of CBD on horses is of particular interest as all cannabinoids are on the list of prohibited substances issued by the international governing body of equestrian sports (FEI, Fédération Equestre Internationale) due to their assumed psychotropic properties (41).

The aim of this study was to analyze stress levels via behavioral observations and heart rate monitoring in healthy horses following oral administration of a CBD containing paste to further validate equine behavior under the influence of CBD medication. The authors hypothesized that increasing CBD doses would have a moderately calming effect in horses.

## 2 Materials and methods

### 2.1 Animals

Twelve Haflinger × Warmblood cross horses, including seven mares and five stallions, were randomly assigned to a treatment or a control group (*n* = 6 + 6). Horses' age varied between 3 to 16 years (median: 11 years) in the treatment group and 10 to 26 years (median: 10.5 years) in the control group. Mares and stallions were housed separately with mares having free paddock access. All horses were fed hay and mineral feed, and spent 8 h a day on pasture. The study was approved by the competent authority for licensing and notification procedures for animal experiments (LAVG) in Brandenburg, Germany (AZ: 2347–12–2021).

### 2.2 Study products

Study products were two pastes (treatment and control). The treatment paste contained 55% full spectrum CBD plant extract, medium-chain triglyceride (MCT) coconut oil, naturally occurring phytocannabinoids, terpenes, flavonoids and beeswax (TAMACAN XL 55%^®^, Herosan healthcare GmbH, Austria). The THC content was below 0.2%. The control paste contained MCT oil and beeswax only. The ingredients of both pastes were analyzed, and concentrations of the contents were confirmed by an independent and internationally accredited anti-doping laboratory (Institute of Biochemistry, German Sport University Cologne, Germany). Pastes were labeled “A” or “B” by the manufacturer before shipment to conceal their formulations. People handling the horses, i.e., caretakers and sample takers, were unaware of the horses' group assignment.

### 2.3 Dose escalation study

The study was divided into three trials with administration of CBD paste in escalating doses (trial 1: 0.2 mg CBD/kg; trial 2: 1 mg CBD/kg; trial 3: 3 mg CBD/kg). Doses were selected based on the manufacturer's recommendation and the current literature (36, 38). The first two trials were performed with three horses in each group (*n* = 3 treatment + 3 control) and close attention was paid to the occurrence of possible side effects. The third trial (3 mg CBD/kg) was subsequently performed with all twelve horses (*n* = 6 treatment + 6 control). The day before each trial, horses were physically examined and a jugular vein catheter was aseptically placed. On the day of trial, the paste (A or B) was orally administered at 6:30 am. For better acceptance, the paste was inserted into a treat. To determine pharmacokinetic parameters of CBD administration in horses, multiple blood and urine samples were taken throughout the trials from all horses (42).

**Figure 1:**
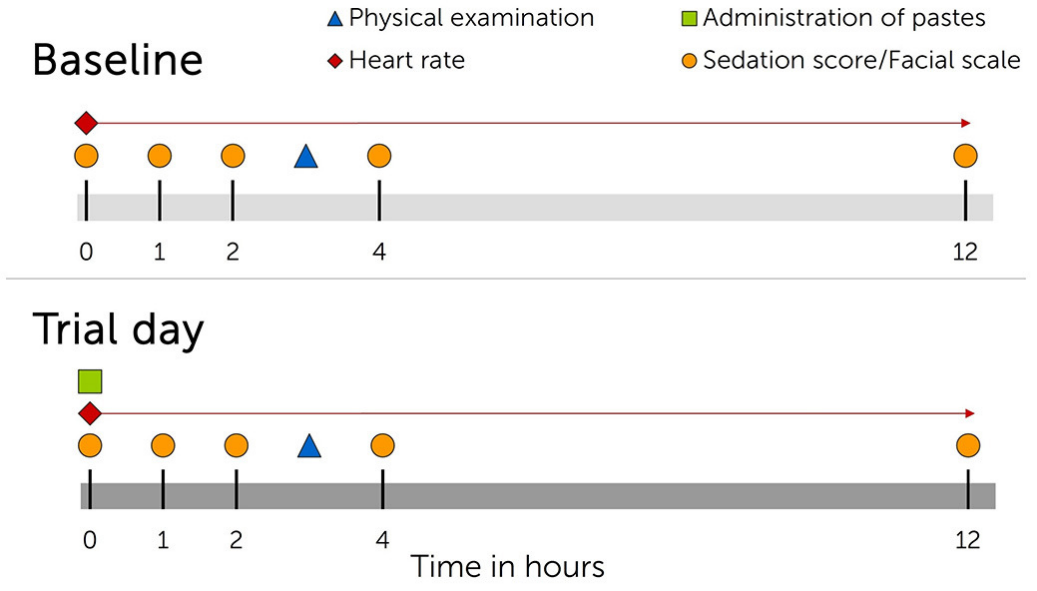
Timeline showing interventions for each cannabidiol (CBD) oral medication trial. **Upper panel**, day before trial start. **Lower panel**, trial day. Trials were repeated three times with single administration of escalating CBD doses (0.2 mg CBD/kg BW; 1 mg CBD/kg BW; 3 mg CBD/kg BW) and wash-out periods of minimum seven days in between trials.

Equine behavior was recorded for the subsequent evaluation of a sedation score by an independent observer at time points 0, 1, 2, 4 and 12 hours (h) after paste administration (Figure 1). The occurrence and the depth of sedation was determined based on the observed position of the horse's head and the reaction to acoustic and visual stimuli (Table 1). Acoustic stimuli included a clicker as it is used for positive reinforcement training as well as the crackling noise of a plastic bag. As a visual stimulus, a pink cloth was attached to a stick and waved in front of the horse's face. Reactions to the stimuli were video recorded. Additionally, photographs were taken for subsequent assessment of the facial expressions. Expressions were rated based on the horse's orbital openings, position of ears, visibility of chewing muscles, position of lips and dilation of nostrils (Table 2).

**Table 1:** Sedation score developed for behavioral observations following single oral administration of cannabidiol (CBD) in three escalating doses (0.2 mg CBD/kg; 1 mg CBD/kg; 3 mg CBD/kg), based on the sedation score by Poller et al. (43).

**Head position**	
1	Lower lip at height of shoulder joint or higher
2	Lower lip between shoulder and olecranon
3	Lower lip between olecranon and carpal joint
4	Lower lip at carpal joint or lower
**Reaction to stimulus: head movement**	
1	Focus directed toward stimulus, jerky aversion
2	Focus directed toward stimulus, aversion, then refocusing on stimulus
3	Focus directed toward stimulus, slight aversion
4	Indifference/no reaction
**Reaction to stimulus: ear movement**	
1	Ears pointed, obvious flickering of ears, steady response to stimulus
2	Moderate flickering of one or both ears
3	Slight flickering of one or both ears
4	Indifference/no reaction
**Reaction to stimulus: Chewing**	
1	Chewing movement is interrupted and does not continue
2	Chewing movement is repeatedly interrupted and recontinued
3	Chewing movement is interrupted once and recontinued
4	Indifference/no interruption of chewing
**Reaction to stimulus: body movement**	
1	Moving back more than one step, turning away
2	Moving back one step, head jerking
3	Jerking/lifting/averting of head
4	Indifference/no reaction
**Total sum for EACH stimulus: 5 - 20**	
**Total sum for ALL stimuli: 15 - 60**	

A total sum was calculated for each stimulus (clicker, bag, cloth) and for all stimuli.

**Table 2:** Facial expression scale developed for behavioral observations following single oral administration of cannabidiol (CBD) paste in three escalating doses (0.2mg CBD/kg; 1mg CBD/kg; 3mg CBD/kg), based on the FaceSed (44) and Horse Grimace Scale (45).

Orbital opening	
2	Eyes completely open
3	Eyes partially open (> 50%)
4	Eyes almost/completely closed (< 50%)
**Position of ears**	
1	Pinned back
2	Forward pointed, position of attention
3	Asymmetrical; one ear hanging
4	Wide opening between ear tips
**Chewing muscles**	
1	Strained/obviously present
2	Moderately present
3	Not present
**Lips**	
1	Strained mouth
2	Loose touching of lips
3	Slight relaxation of one lip
4	Pronounced relaxation/hanging of one lip
**Nostrils**	
1	Dilated, outer ring clearly visible
2	Non-dilated nostrils
3	Small nostrils, relaxed outer ring
**Total sum: 6 - 18**	

Each horse's heart rate (HR) was continuously recorded throughout the trials using a Polar^®^ H10 heart rate sensor (Polar^®^ Electro Oy, Kempele, Finland). The sensor was attached to an electrode belt which spanned around the horse's chest. To enhance skin contact and signal transmission, the coat was trimmed and moisturized with water over the heart base between the 4^th^ and 5^th^ intercostal space where the electrodes were positioned. Each sensor was connected to a mobile device via Bluetooth to document the cardiac beat-to-beat (R-R) intervals with the Polar^®^ Equine App (Version 1.2.1, Polar^®^ Electro, Kempele, Finland).

Repeated physical examination was performed 2–4 h following paste administration, and blood samples were obtained for white blood cell (WBC) count.

Baseline values including recordings of equine behavior and heart rate were obtained in the same pattern as described on the day before each trial for comparative analysis (Figure 1). Trials were divided by wash-out periods of at least seven days.

### 2.4 Assessment of behavioral observations

Evaluation of the video recordings was based on a previously described sedation score (43). For assessment of the photographs, a facial expression scale was developed based on the facial sedation scale for horses (FaceSed) (44) and the Horse Grimace Scale (45). The described parameters were modified according to the reactions and expressions observed in the study animals (Tables 1, 2). Videos and photographs of each horse were randomly arranged and blinded assessment was performed by one person who was experienced in equine behavior studies but not actively involved in any of the trials. For each horse, stimulus and time point, the five parameters of the sedation score were summed up, resulting in scores ranging from 5 to 20 (Table 1). The scores of the three stimuli were then summed up to a total for each horse and time point, resulting in a total sedation score ranging from 15 to 60. For the facial expression scale, parameters were similarly added up to a possible total sum of 6–18 for each time point and each individual horse. A score of 10 was given when the eyes were open, the ears forward pointing, the chewing muscles moderately present, the lips loosely touching and the nostrils non-dilated (Table 2). High scores represent a deeper relaxation or sedation.

### 2.5 Assessment of heart rate and heart rate variability

Heart rate (HR) and heart rate variability (HRV) were analyzed using the software Kubios^®^ HRV Standard (ver. 3.5, Kubios^®^ Oy, Kuopio, Finland). Parameters included the mean HR in beats per minute (bpm), the root mean square of successive beat-to-beat differences (RMSSD in milliseconds, ms) and the standard deviation of normal-to-normal beat-to-beat intervals (SDNN, ms). Automatic beat correction was applied to remove artifacts (threshold: very low, 0.3 s). Each recording period was divided into sections of 15 min as previously described (46).

### 2.6 Statistical analysis

Data were recorded in Microsoft Excel^®^ (Version 2304) and statistical analysis was performed with SPSS^®^ Statistics 27 (IBM^®^, NY, USA). First, data was analyzed descriptively: The value for each total sedation score and the sedation scores of the three stimuli were displayed in bar charts (mean + standard deviation). For the inductive analysis, the difference between the total sedation score at baseline and during the trial was calculated for each horse and time point (ranging from −45 to +45). Similarly, the differences between score on baseline and trial day were calculated for the facial expression scale (ranging from −12 to +12). The effects of the dose levels on the differences between baseline and trial day of the total sedation score were analyzed using linear mixed models. Individual horses were assigned as subjects, dose levels as fixed effects (reference = control group; trial 1 = 0.2 mg CBD/kg; trial 2 = 1 mg CBD/kg; trial 3 = 3 mg CBD/kg) and time points as random effects (0 h; 1 h; 2 h; 4 h; 12 h). Residuals were visually inspected for normal distribution. The level of significance was *p* < 0.05. For the facial expression scale, the differences between baseline and trial day were calculated and tested for an effect of dose levels using a linear mixed model as described above.

For HR, RMSSD and SDNN parameters, the first eight 15-minute sections (total of two hours) post paste administration were selected for analysis as CBD blood concentrations reached a maximum here (42). To test for an effect of dose levels on the parameters, linear mixed models were calculated as described above.

To identify systematic differences between baseline and trial day values of HR, RMSSD and SDNN within the treatment group over time, linear mixed models for each outcome were calculated with trials (reference = baseline; trial 1 = 0.2 mg CBD/kg; trial 2 = 1 mg CBD/kg; trial 3 = 3 mg CBD/kg) as fixed effects. The following analysis was performed as described above with individual horses as subjects, dose levels as fixed effects and time points as random effects.

## 3 Results

### 3.1 Animals

The horses' body weight was on average 488 ± 55 kg in the treatment group and 443 ± 56 kg in the control group. During the first two trials, no side effects such as gastrointestinal intolerances were observed following paste application and it was considered safe to proceed with trial three. During trial three, one mare developed signs of a jugular vein thrombophlebitis and was excluded, resulting in five remaining horses in the treatment group to complete trial three (*n* = 5 + 6). Over all trials, the WBC count remained close to reference range with only mild WBC elevation (maximum WBC in the treatment group = 11.63 10^9^/L) (Table 3).

**Table 3:** Mean ± standard deviation of white blood cell (WBC) count after single oral administration of a cannabidiol (CBD) containing paste in three trials.

**Parameter (Ref)**	**First trial (0.2 mg CBD/kg)**	**Second trial (1 mg CBD/kg)**	**Third trial (3 mg CBD/kg)**
**Control group**			
WBC count (5–10 10^9^/L)	7.43 ± 0.98	6.88 ± 0.38	7.79 ± 1.28
Number of horses out of Ref (Value out of Ref)	*n =* 0/3	*n =* 0/3	*n =* 1/6 (10.31 10^9^/L)
**Treatment group**			
WBC count (5–10 10^9^/L)	10.49 ± 0.68	9.79 ± 1.33	7.97 ± 2.19
Number of horses out of Ref (Value out of Ref)	*n =* 1/3 (11.17 10^9^/L)	*n =* 1/3 (11.63 10^9^/L)	*n =* 1/5 (11.60 10^9^/L)

The number of horses with serum levels outside of the reference range (Ref) are reported for each group.

### 3.2 Behavioral observations

#### 3.2.1 Sedation score

**Figure 2:**
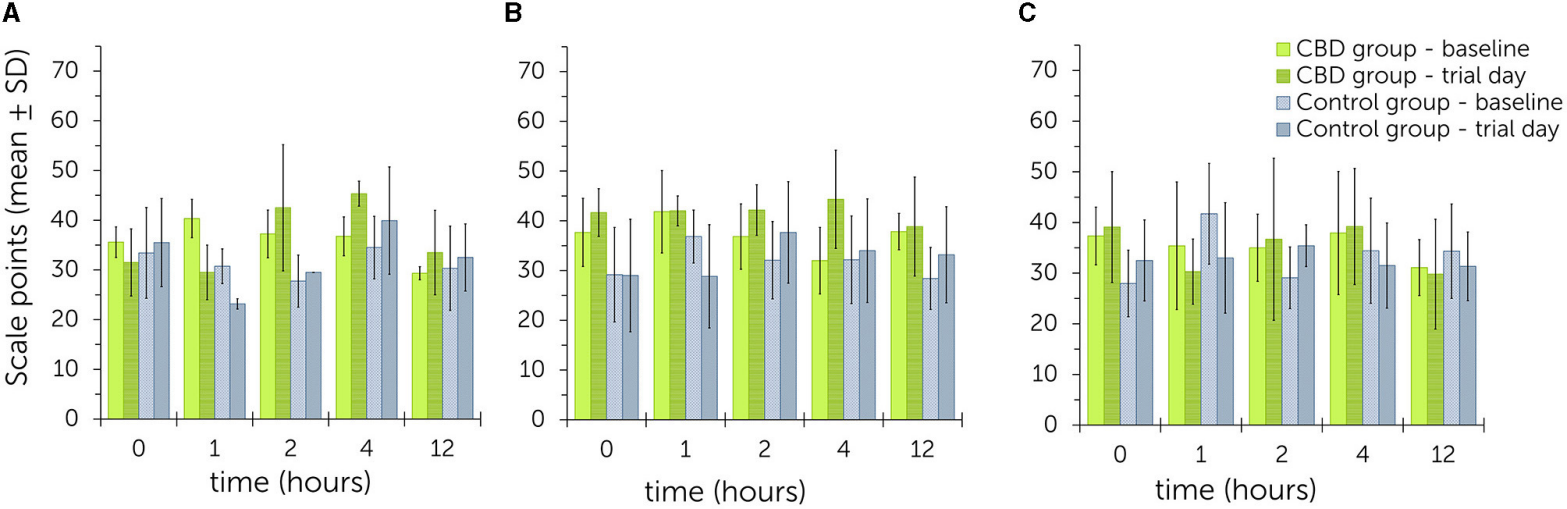
Summed up sedation scores after acoustic and visual stimulations (clicker, plastic bag, pink cloth) following single oral administration of cannabidiol (CBD) paste in escalating doses (**A**: 0.2 mg CBD/kg; **B**: 1 mg CBD/kg; **C**: 3 mg CBD/kg) - comparison between values obtained on baseline and trial day for the treatment and control group. Higher scale points relate to a higher level of sedation (Table 1). No significant differences were found between treatment and control group over all three trials.

For all three trials, graphical illustration of the statistical data using bar charts did not identify a clear trend for higher or lower sedation scores between groups or dose levels (Figure 2, Supplementary Figures S1–S3). During trial 1, overall scores for baseline values ranged from 29.3 ± 1.3 to 40.3 ± 3.9 at all time points in the treatment group. Overall scores for trial day values ranged from 29.5 ± 5.5 to 45.3 ± 2.5 at all time points. In the control group, values ranged between 27.8 ± 5.3 to 34.5 ± 6.3 at baseline and between 23.2 ± 1.0 to 39.9 ± 10.8 on trial day. No trend was observed for values being generally higher or lower at certain time points in either group.

During trial 2, baseline values ranged from 32.0 ± 6.7 to 41.8 ± 8.3 and trial day values from 38.8 ± 10.0 to 44.3 ± 9.9 in the treatment group. All values were higher on trial day than at baseline as exemplified by graphical illustration. In the control group, baseline values were between 28.4 ± 6.2 to 36.8 ± 7.3 and trial day values between 28.8 ± 10.4 to 37.7 ± 10.2. Values were higher on trial day than the corresponding baseline values at time points 2, 4 and 12.

During trial 3, baseline values in the treatment group were between 31.1 ± 5.5 to 37.9 ± 12.2 and trial day values between 29.8 ± 10.8 to 39.2 ± 11.4. In the control group, baseline values ranged from 28.0 ± 6.6 to 41.7 ± 9.9 and trial day values from 31.3 ± 6.7 to 35.4 ± 4.1. No trend was observed for values being generally higher or lower at certain time points in either group.

Linear mixed models with escalating doses as fixed effects did not identify significant differences between the total sum of sedation scores in the treatment and control group [*P*(*F*) = 0.527]. Even during trial 2, the difference was not significant [*P*(*F*) = 0.180]. Similarly, the individual scores were not significantly influenced by escalating doses for stimulation with a clicker [*P*(*F*) = 0.196], crackling of a plastic bag [*P*(*F*) = 0.442] or waving with the pink cloth [*P*(*F*) = 0.915]. Estimates for random effects for the total sum were: β = 25.9 [95% confidence intervals (CI) = 6.7, 100.6; standard error (*SE*) = 17.9], for clicker: β = 7.7 (95% CI = 2.9, 20.4; *SE* = 3.8) and for plastic bag: β = 1.3 (95% CI = 0.0, 126.8; *SE* = 3.0). Random effects were not estimated for visual stimulation with a cloth. For the total sum, 21.7% of variability was accounted to differences between time points. For stimulation with a clicker and plastic bag, time points as random effects were attributed to 32.6 and 4.7% of variability, respectively.

#### 3.2.2 Facial expression scale

**Figure 3:**
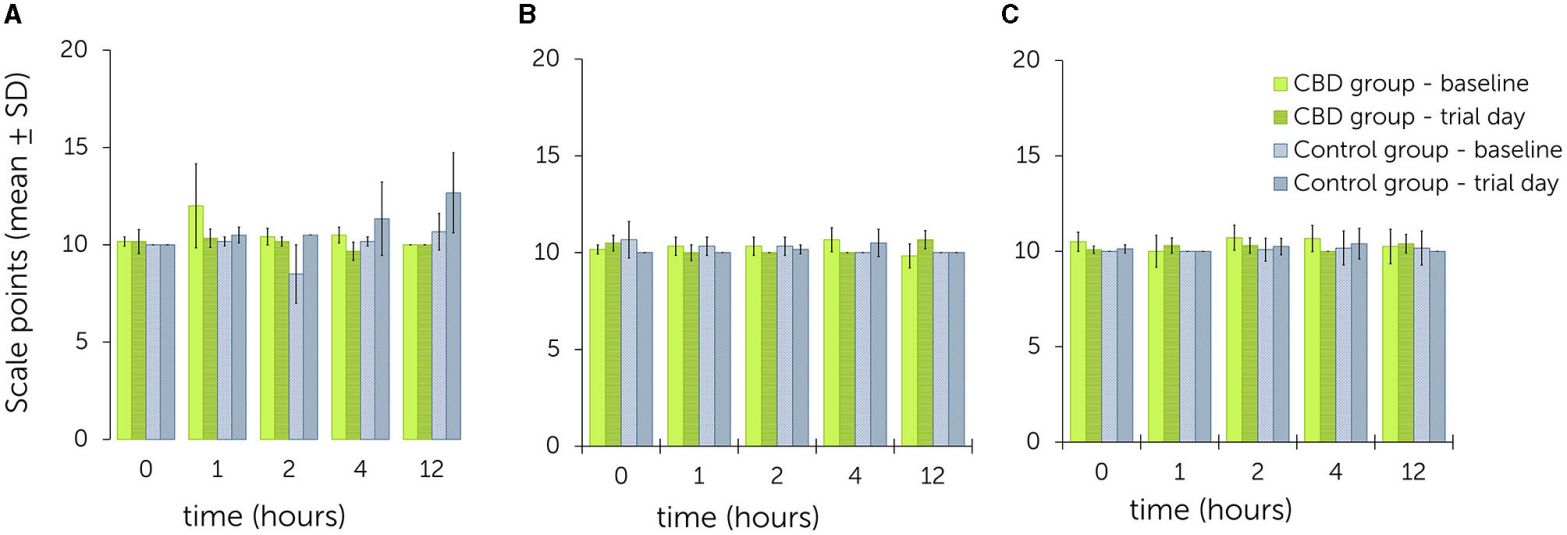
Facial expression scale following single oral administration of cannabidiol (CBD) paste in escalating doses (**A**: 0.2 mg CBD/kg; **B**: 1 mg CBD/kg; **C**: 3 mg CBD/kg) - comparison between values obtained on baseline and trial day for the treatment and control group. Higher scale points relate to a higher level of sedation (Table 2).

Examples for scoring of the facial expressions are shown in Supplementary Table S1. Graphical illustration of sedation scores is shown in Figure 3.

During trial 1, overall scores for baseline values ranged from 10.0 ± 0.0 to 12.0 ± 2.2 at all time points in the treatment group. Overall scores for trial day values ranged from 9.7 ± 0.5 to 10.3 ± 0.5 at all time points. All values were equal or lower on trial day than at baseline. In the control group, baseline values ranged from 8.5 ± 1.5 to 10.7 ± 0.9 and from 10.0 ± 0.0 to 12.7 ± 2.1 on trial day. All values were equal or higher on trial day than at baseline. In this trial, the most notable differences between baseline and trial day were found at time point 1 (treatment group: 12.0 ± 2.2 to 10.3 ± 0.5) and time point 12 (control group: 10.7 ± 0.9 to 12.7 ± 2.1).

During trial 2, baseline values in the treatment group were between 9.8 ± 0.6 to 10.7 ± 0.6 and trial day values between 10.0 ± 0.0 to 10.7 ± 0.5. In the control group, baseline values ranged from 10.0 ± 0.0 to 10.7 ± 0.9 and trial day values from 10.0 ± 0.0 to 10.5 ± 0.7. No trend was observed for values being generally higher or lower at certain time points in either group.

During trial 3, baseline values in the treatment group ranged from 10.0 ± 0.8 to 10.7 ± 0.7 and trial day values from 10.0 ± 0.0 to 10.4 ± 0.5. In the control group, baseline values ranged from 10.0 ± 0.0 to 10.2 ± 0.9 and trial day values from 10.0 ± 0.0 to 10.4 ± 0.8. No trend was observed for values being generally higher or lower at certain time points in either group.

The linear mixed model did not identify a significant effect of escalating CBD doses on the facial expression scale when compared to the control group [*P*(*F*) = 0.080]. Considering the fixed effects estimates, a significant effect was evident between trial 1 and the control group (*p* = 0.021) (Table 4). The estimate for the random effects was β = 0.1 (95% CI = 0.0, 27.4; *SE* = 0.2) with 3.3% of variability attributed to differences between time points.

**Table 4:** Fixed effects estimates for the comparison of differences (Δ) between score levels reached on a facial expression scale on baseline and trial days [single oral administration of cannabidiol (CBD) paste in three escalating doses (0.2 mg CBD/kg; 1 mg CBD/kg; 3 mg CBD/kg)].

**Parameter**	**Regression coefficient (β)**	**95% confidence intervals (CI)**	**Standard error (SE)**	***p-*value**
Δ **Score levels (facial expression scale)**				
Intercept	0.3	0.0, 0.7	0.2	0.077
Control group	Reference		
Trial 1 (0.2 mg CBD/kg)	−0.9	−1.6, −0.1	0.4	0.021
Trial 2 (1 mg CBD/kg)	−0.4	−1.1, 0.4	0.4	0.344
Trial 3 (3 mg CBD/kg)	−0.6	−1.2, 0.0	0.3	0.065

### 3.3 Heart rate and heart rate variability

#### 3.3.1 Comparison between treatment and control group

Mean HR and HRV values are shown in Table 5. On trial days, the mean HR in the first 2 h post paste administration was between 42.1 ± 8.6 bpm to 45.4 ± 7.5 bpm in the treatment group, and between 41.3 ± 8.2 bpm to 44.4 ± 9.8 bpm in the control group.

**Table 5:** Mean ± SD values for HR, RMSSD and SDNN values from the first 2 h after single oral cannabidiol (CBD) paste administration with corresponding baseline values. Due to technical issues, the trial 1 R-R-interval data are partly incomplete.

**Parameter**	**Treatment group – baseline (mean ±SD)**	**Treatment group – trial day (mean ±SD)**	**Control group – baseline (mean ±SD)**	**Control group – trial day (mean ±SD)**
**HR (bpm)**				
Trial 1 (0.2 mg CBD/kg)	30.2 ± 2.9	45.4 ± 7.5	no data	41.4 ± 4.6
Trial 2 (1 mg CBD/kg)	45.3 ± 7.0	43.3 ± 4.1	43.2 ± 7.2	41.3 ± 8.2
Trial 3 (3 mg CBD/kg)	42.6 ± 6.6	42.1 ± 8.6	39.0 ± 4.4	44.4 ± 9.8
**RMSSD (ms)**				
Trial 1 (0.2 mg CBD/kg)	127.7 ± 51.2	152.9 ± 36.6	no data	151.6 ± 29.3
Trial 2 (1 mg CBD/kg)	112.7 ± 33.8	123.6 ± 30.6	151.3 ± 39.4	137.1 ± 35.4
Trial 3 (3 mg CBD/kg)	113.8 ± 40.0	122.7 ± 48.8	151.0 ± 61.7	140.9 ± 48.2
**SDNN (ms)**				
Trial 1 (0.2 mg CBD/kg)	140.8 ± 44.6	163.1 ± 48.4	no data	156.8 ± 49.6
Trial 2 (1 mg CBD/kg)	110.1 ± 41.0	105.4 ± 22.8	154.4 ± 71.1	146.0 ± 49.7
Trial 3 (3 mg CBD/kg)	104.6 ± 44.7	131.0 ± 61.1	121.5 ± 38.5	135.7 ± 64.4

SD, standard deviation; HR, heart rate; RMSSD, root mean square of successive R-R interval differences; SDNN, standard deviation of normal-to-normal R-R intervals; bpm, beats per minute; ms, milliseconds.

**Figure 4:**
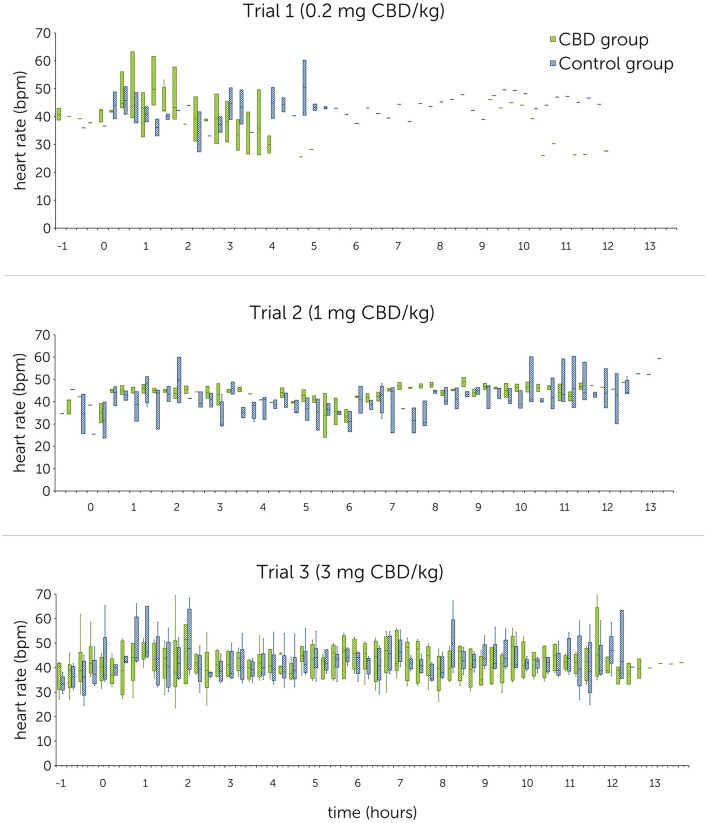
Heart rates [beats per minute (bpm)] following single oral administration of cannabidiol (CBD) in three escalating doses (0.2 mg CBD/kg BW; 1 mg CBD/kg BW; 3 mg CBD/kg BW) at time point 0, displayed in 15-min sections over 12 h. Due to technical issues, the trial 1 R-R-interval data are partly incomplete.

**Figure 5:**
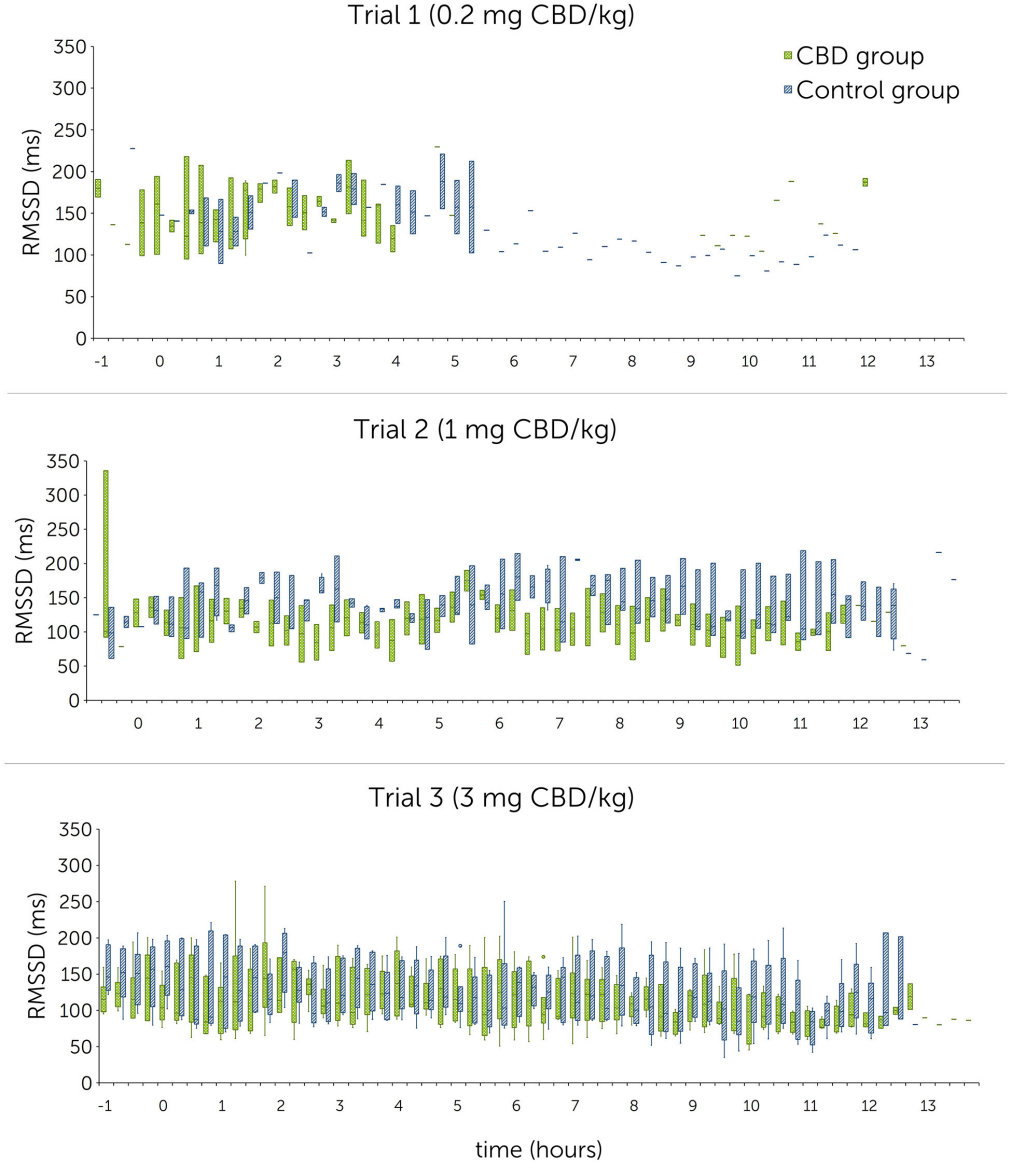
Root mean square of successive R-R interval differences (RMSSD) in milliseconds (ms) following single oral administration of cannabidiol (CBD) in three escalating doses (0.2 mg CBD/kg BW; 1 mg CBD/kg BW; 3 mg CBD/kg BW) at time point 0, displayed in 15-min sections over 12 h. Due to technical issues, the trial 1 R-R-interval data are partly incomplete.

**Figure 6:**
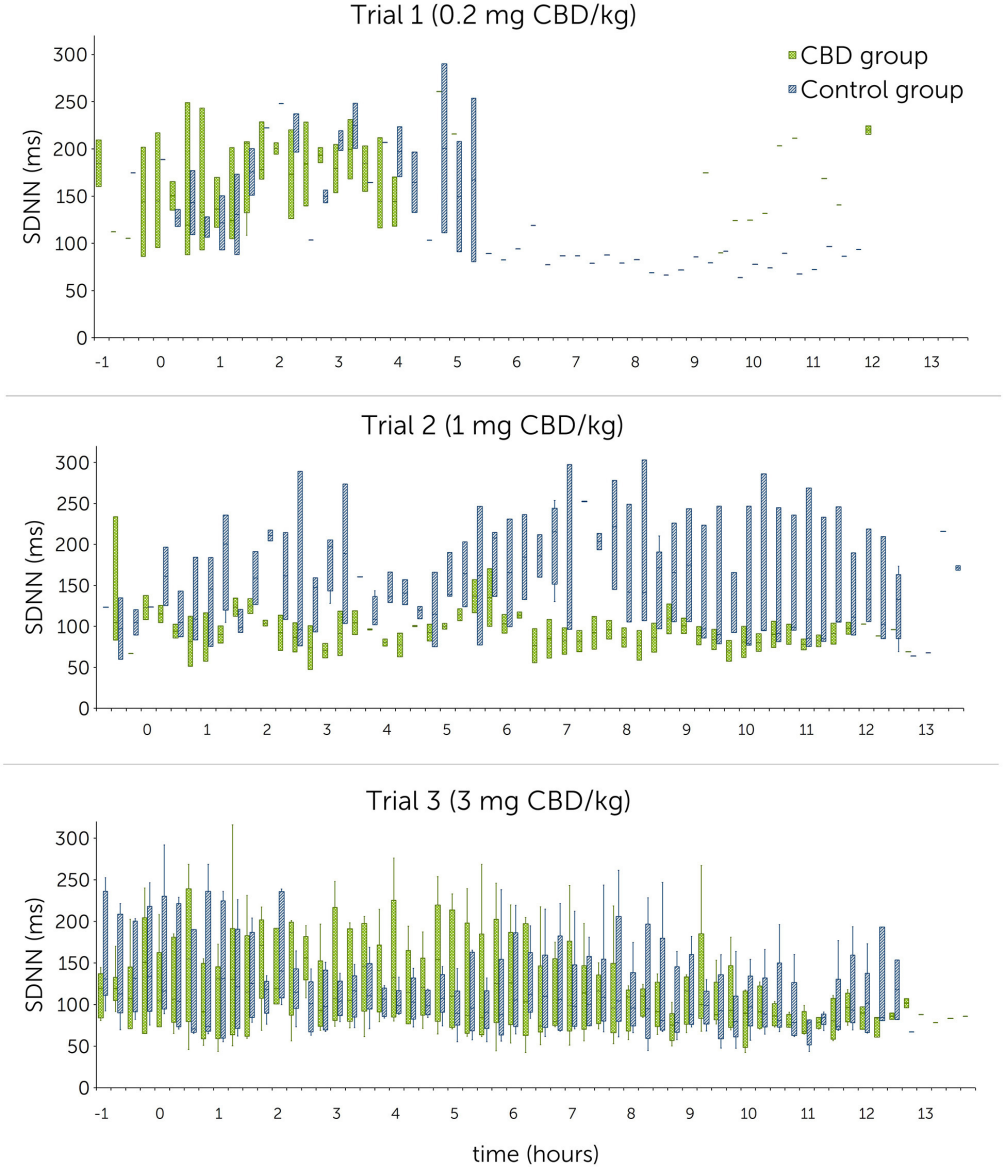
Normal-to-normal R-R intervals (SDNN) in milliseconds (ms) following single oral administration of cannabidiol (CBD) in three escalating doses (0.2 mg CBD/kg BW; 1 mg CBD/kg BW; 3 mg CBD/kg BW) at time point 0, displayed in 15-min sections over 12 h. Due to technical issues, the trial 1 R-R-interval data are partly incomplete.

RMSSD values ranged between 122.7 ± 48.8 ms and 152.9 ± 36.6 ms in the treatment group, and 137.1 ± 35.4 ms and 151.6 ± 29.3 ms in the control group. For SDNN, mean values were between 105.4 ± 22.8 ms and 163.1 ± 48.4 ms in the treatment group, and between 135.7 ± 64.4 ms and 156.8 ± 49.6 ms in the control group. Graphical representations of mean HR, RMSSD and SDNN are shown in Figures 4–6 (trial days) and Supplementary Figures S4–S6 (baseline).

Statistical analysis using linear mixed models found that doses as fixed effects had no significant impact on HR [*P*(*F*) = 0.139], RMSSD [*P*(*F*) = 0.104] and SDNN [*P*(*F*) = 0.202]. A significant difference could not be identified even between the highest CBD dose (3 mg CBD/kg) and the control group (HR: *p* = 0.377; RMSSD: *p* = 0.189; SDNN: *p* = 0.734) (Table 6).

**Table 6:** Fixed effects estimates for comparison between treatment and control group of HR, RMSSD and SDNN values from the first 2 h following single oral administration of cannabidiol (CBD) paste in three escalating doses (0.2mg CBD/kg; 1mg CBD/kg; 3mg CBD/kg).

**Parameter**	**Regression coefficient (β)**	**95% confidence intervals (CI)**	**Standard error (SE)**	***p-*value**
**HR (bpm)**				
Intercept	43.7	41.4, 46.0	1.1	< 0.001
Control group	Reference			
Trial 1 (0.2 mg CBD/kg)	2.6	−1.4, 6.5	2.0	0.196
Trial 2 (1 mg CBD/kg)	0.5	−4.1, 5.1	2.3	0.826
Trial 3 (3 mg CBD/kg)	−1.5	−4.8, 1.8	1.7	0.377
**RMSSD (ms)**				
Intercept	134.6	123.4, 145.8	5.6	< 0.001
Control group	Reference			
Trial 1 (0.2 mg CBD/kg)	11.6	−8.3, 31.6	10.1	0.251
Trial 2 (1 mg CBD/kg)	2.9	−20.5, 26.2	11.8	0.809
Trial 3 (3 mg CBD/kg)	−11.0	−27.5, 5.5	8.3	0.189
**SDNN (ms)**				
Intercept	135.8	120.7, 150.8	7.5	< 0.001
Control group	Reference			
Trial 1 (0.2 mg CBD/kg)	18.1	−8.7, 44.9	13.5	0.184
Trial 2 (1 mg CBD/kg)	−12.1	−43.3, 19.1	15.8	0.445
Trial 3 (3 mg CBD/kg)	−3.8	−26.0, 18.4	11.2	0.734

HR, heart rate; RMSSD, root mean square of successive R-R interval differences; SDNN, standard deviation of normal-to-normal R-R intervals; bpm, beats per minute; ms, milliseconds.

For HR, the estimate for the random effects was β = 31.5 (95% CI = 15.1, 65.7; *SE* = 11.8). Differences between time sections are accounted for 44.1% of variability. The RMSSD estimate was β = 607.0 (95% CI = 262.0, 1406.3; *SE* = 260.2) and 33.2% of variability was attributed to time sections. For SDNN, β was 1107.0 (95% CI = 456.3, 2685.8; *SE* = 500.6). Time sections were associated with 33.7% of variability.

#### 3.3.2 Comparison between baseline and trial day within the treatment group

Mean HR values showed no trend indicating a consistent increase or decrease from baseline to trial day in the treatment group (Table 5). Mean RMSSD and SDNN values showed a consistent increase from baseline to trial day during all trials, except for a decrease in SDNN values during trial 2 (110.1 ± 41.0 ms to 105.4 ± 22.8 ms).

Examination of the differences between baseline and trial day values identified no significant effect for HR [*P*(*F*) = 0.136] over all three trials but found significant effects for RMSSD [*P*(*F*) = 0.016] and SDNN [*P*(*F*) < 0.001]. Both significant findings can be attributed to trial 1 and trial 3 (Table 7). Estimates for random effects for HR were: β = 13.1 (95% CI = 5.0, 34.1; *SE* = 6.4), for RMSSD: β = 768.5 (95% CI = 399.6, 1478.2; *SE* = 256.5) and for SDNN: β = 1052.6 (95% CI = 537.88, 2060.1; *SE* = 360.6). For HR, RMSSD and SDNN values, differences between time sections are accounted for 22.5%, 40.6% and 39.6% of variability, respectively.

**Table 7:** Fixed effects estimates for comparison within the treatment group of HR, RMSSD and SDNN values from the first 2 h between baseline and following single oral administration of cannabidiol (CBD) paste in three escalating doses (0.2mg CBD/kg; 1mg CBD/kg; 3mg CBD/kg).

**Parameter**	**Regression coefficient (β)**	**95% confidence intervals (CI)**	**Standard error (SE)**	***p-*value**
**HR (bpm)**				
Intercept	42.5	40.6, 44.4	1.0	< 0.001
Baseline values	Reference			
Trial 1 (0.2 mg CBD/kg)	3.4	0.3, 6.6	1.6	0.034
Trial 2 (1 mg CBD/kg)	0.9	−2.7, 4.5	1.8	0.627
Trial 3 (3 mg CBD/kg)	−0.4	−2.9, 2.1	1.3	0.766
**RMSSD (ms)**				
Intercept	118.4	107.2, 120.5	5.6	< 0.001
Baseline values	Reference			
Trial 1 (0.2 mg CBD/kg)	25.0	8.8, 41.1	8.2	0.003
Trial 2 (1 mg CBD/kg)	16.6	−1.8, 35.1	9.3	0.077
Trial 3 (3 mg CBD/kg)	7.7	−5.1, 20.5	6.5	0.233
**SDNN (ms)**				
Intercept	112.4	99.2, 125.6	6.6	< 0.001
Baseline values	Reference			
Trial 1 (0.2 mg CBD/kg)	40.1	20.8, 59.4	9.8	< 0.001
Trial 2 (1 mg CBD/kg)	3.0	−19.0, 25.1	11.1	0.785
Trial 3 (3 mg CBD/kg)	21.3	6.0, 36.6	7.7	0.007

HR, heart rate; RMSSD, root mean square of successive R-R interval differences; SDNN, standard deviation of normal-to-normal R-R intervals; bpm, beats per minute; ms, milliseconds.

## 4 Discussion

Investigation of stress parameters in healthy horses, including behavioral observations and heart rate monitoring, following oral administration of a CBD containing paste in escalating doses did not identify consistently significant differences when compared to a control group.

CBD products are marketed for a variety of conditions in animals including improving general wellbeing and having a calming and stress-relieving effect (3–8). Sedation is a reported side effect associated with CBD application in humans and dogs (30–34, 47). To assess sedation in horses, multiple scoring systems have been proposed but are mainly aimed at testing sedatives such as detomidine or acepromazine (43, 48, 49). As levels of sedation in this study were not pronounced and scoring based on established scales did not produce satisfying results, a previously described sedation scale (43) was adjusted to the behavior exhibited by the horses in the current study (37). The dose levels tested in this study (0.2 mg CBD/kg, 1 mg CBD/kg, 3 mg CBD/kg) did not result in any significant difference in sedation scores after acoustic or visual stimulation compared to the control group. This is in agreement with a previous report where sedation levels were scored in horses following CBD administration (37). In this report, pellets containing 150 mg CBD (~ 0.29 mg CBD/kg) were fed over 56 days with no significant difference in sedation levels detected when compared to a control group. In humans, sedation was described as a side effect after daily oral intake of a total of 600 mg CBD over 6 weeks (47). Future studies may investigate whether higher dose administrations lead to more significant signs of sedation in horses.

Photographs were taken to assess the potential influence of CBD on equine facial expression. Existing scoring systems including FaceSed and Horse Grimace Scale (HGS) were modified to suit the purpose of the current report, as CBD administration did not produce sedation levels comparative to those depicted in the FaceSed scale (44, 45). Horses additionally displayed facial expressions described in the HGS, like strained mouth and chewing muscles. As the horses included in the current study did not undergo any painful procedures, similar expressions were interpreted as signs of stress. Expressions related to annoyance, such as pinned-back ears, were also exhibited. Only the modified scores of trial 1 (0.2 mg CBD/kg) were significantly different when compared between treatment and control group (*p* = 0.021). Score levels were higher at baseline than on trial day in the treatment group at time points 1, 2 and 4, whereas score levels in the control group were consistently lower at baseline than on trial day (Figure 3). As this result is the only significant event in this study part and comparisons with higher dose administrations did not produce significant results, its relevance should be interpreted with caution.

CBD reduces anxiety and stress by acting as a direct or indirect agonist on 5-HT_1A_- and CB_1_-receptors (10–14, 20). Stress levels can be evaluated based on changes of heart rate and heart rate variability in horses (50–53). A comparatively lower HR and increased HRV values (RMSSD and SDNN) indicate an autonomic shift toward a parasympathetic dominance and therefore a reduction of stress (50, 52, 54). In rodents, one-time intraperitoneally injected CBD (10 mg/kg) has been shown to reduce the increase of HR and blood pressure in a stress inducing and fear conditioning setting, suggesting an anxiolytic effect similar to diazepam (24, 55). Another study identified a modest effect of oral CBD (total dose: 30 mg) on resting HR and HRV in humans (29). The relevance for physiological functions with the shown effect is however questionable and should be evaluated with caution as the study design did not include a control group (29). Other studies in horses and dogs showed no influence of CBD on HR or HRV so far: One study in horses found no significant difference in HR during a novel object test between a treatment group fed 100 mg pelleted CBD (~ 0.2 mg CBD/kg) and a control group (36). In dogs, a treatment and a placebo group displayed similar HR and HRV values during a stress test. The dose tested here was 4 mg CBD/kg, administered orally every day over a period of 6 months (56). Similarly, dogs treated orally with 1.4 mg CBD/kg showed no significant changes in RMSSD and SDNN following a fear response test (57). To the best of the authors' knowledge, there are no studies investigating the effect of CBD on resting HR and HRV in healthy horses so far. Due to the short interval of stimulation, it was decided not to specifically analyze HR and HRV during sedation scoring including acoustic and visual stimuli in the current study. HR and HRV compared over the first 2 h after paste administration identified non-significant differences between the treatment and control group in all trials. Comparison within the treatment group showed a consistent increase of the RMSSD compared between all three baseline and trial day values with a significant effect identified for trial 1 (0.2 mg CBD/kg) (Table 7). For SDNN, significant increases were detected for trial 1 and trial 3 (3 mg CBD/kg) (Table 7). These results point toward a decreased sympathetic and an increased parasympathetic tonus following CBD administration and support the hypothesized relaxing effect of CBD. However, as the 95% confidence intervals are large, results should still be interpreted with caution.

Cannabis and cannabinoids are FEI declared prohibited substances, with CBD and cannabidiolic acid (CBDA) listed as controlled medication, due to their possible psychotropic and analgesic properties (41). In this study, an influence of CBD in escalating dose levels on equine behavioral parameters could not be confirmed, but it cannot be excluded that higher doses or administration over longer time periods would influence a horse's behavior. As horses in the current study were healthy and displayed a calm behavior throughout, the effect of CBD on stressed or anxious horses would be an additional point of interest.

Limitations of this study include the small sample size and the assessment of single administrations of one CBD containing product only. As horses were closely monitored and sedation levels were scored multiple times per day, a habituation effect cannot be excluded. Signs of stress or annoyance as evident on the photographs may partially result from repeated testing. However, as treatment and control groups underwent the exact same protocol, the effect of repeated testing was deemed negligible as it was concluded that it would have occurred similarly in both groups.

## 5 Conclusions

The analysis of stress parameters did not identify consistently significant effects of orally administered CBD on levels of sedation, the resting heart rate or heart rate variability in horses. Escalating doses (0.2 mg CBD/kg to 3 mg CBD/kg) did not result in a significant reduction of the heart rate, or increased sedation or relaxation. Oral administration of CBD containing paste proved to be well-tolerated and did not cause any side effects. Further research is required to determine specific indications for the use of CBD products in horses.

## Data availability statement

The raw data supporting the conclusions of this article will be made available by the authors, without undue reservation.

## Ethics statement

The animal study was approved by the authority for licensing and notification procedures for animal experiments (LAVG) in Brandenburg, Germany (AZ: 2347–12–2021). The study was conducted in accordance with the local legislation and institutional requirements.

## Author contributions

FE: Conceptualization, Data curation, Formal analysis, Investigation, Methodology, Project administration, Software, Validation, Visualization, Writing—original draft. AE: Conceptualization, Data curation, Formal analysis, Funding acquisition, Investigation, Methodology, Project administration, Resources, Supervision, Validation, Writing—review & editing. KCJ: Formal analysis, Methodology, Software, Validation, Writing—review & editing. NB: Conceptualization, Data curation, Methodology, Project administration, Writing—review & editing. HP: Data curation, Formal analysis, Investigation, Methodology, Writing—review & editing. WB: Conceptualization, Methodology, Project administration, Supervision, Writing—review & editing. CL: Conceptualization, Funding acquisition, Methodology, Project administration, Resources, Supervision, Writing—review & editing. MW: Conceptualization, Investigation, Methodology, Project administration, Resources, Supervision, Writing—review & editing.
