# Supplementary material for: Behavioral observations, heart rate and heart rate variability in horses following oral administration of a cannabidiol containing paste in three escalating doses (part 1/2)
Source: Front Vet Sci. 2023 Dec 11;10:1305868. doi: 10.3389/fvets.2023.1305868 (PMC10750369; doi:10.3389/fvets.2023.1305868)
Supplement: Supplementary file 1 [file Data_Sheet_1.pdf]

SUPPLEMENTARY MATERIAL

1 Figures

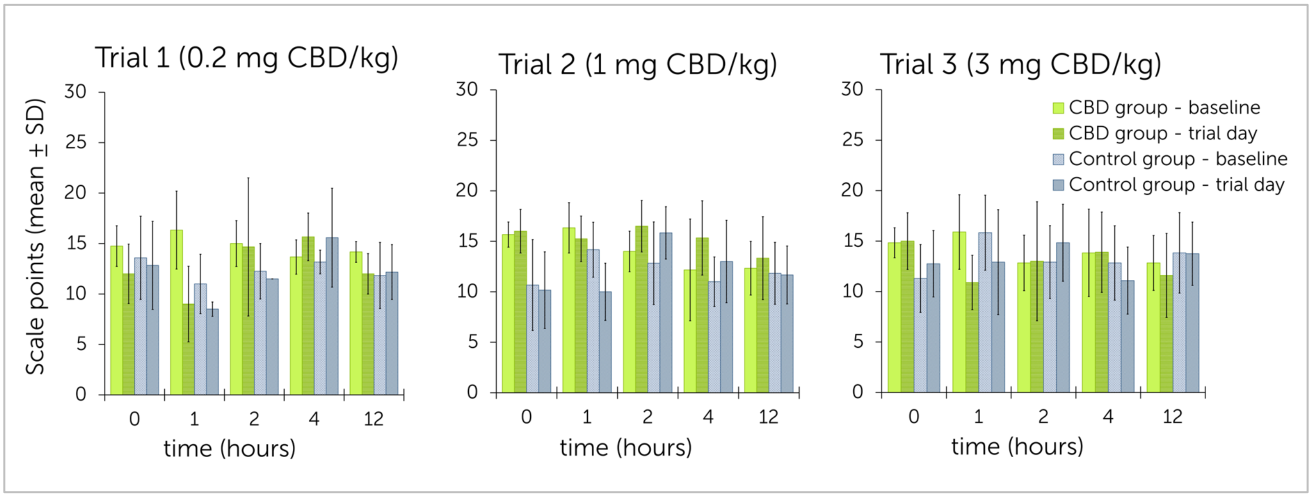

Supplementary Figure 1S: Sedation scores after acoustic stimulation with a clicker following single oral administration of cannabidiol (CBD) paste in escalating doses (0.2 mg CBD/kg; 1 mg CBD/kg; 3 mg CBD/kg) - comparison between values obtained on baseline and trial day for the treatment and control group. Higher scale points relate to a higher level of sedation (Table 1).

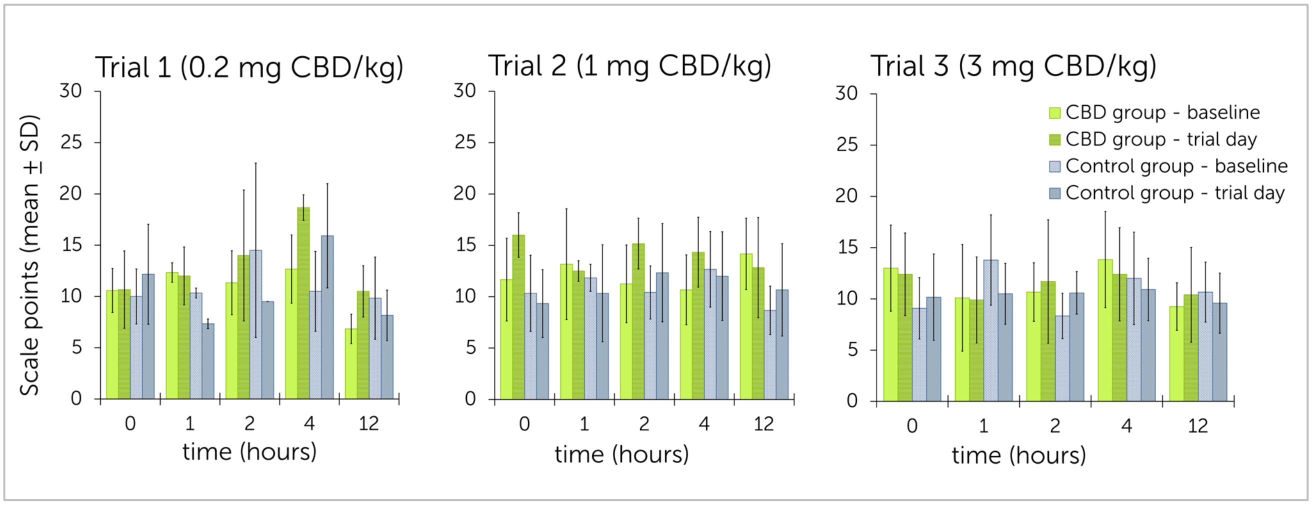

Supplementary Figure 2S: Sedation scores after acoustic stimulation with a plastic bag following single oral administration of cannabidiol (CBD) paste in escalating doses (0.2 mg CBD/kg; 1 mg CBD/kg; 3 mg CBD/kg) - comparison between values obtained on baseline and trial day for the treatment and control group. Higher scale points relate to a higher level of sedation (Table 1).

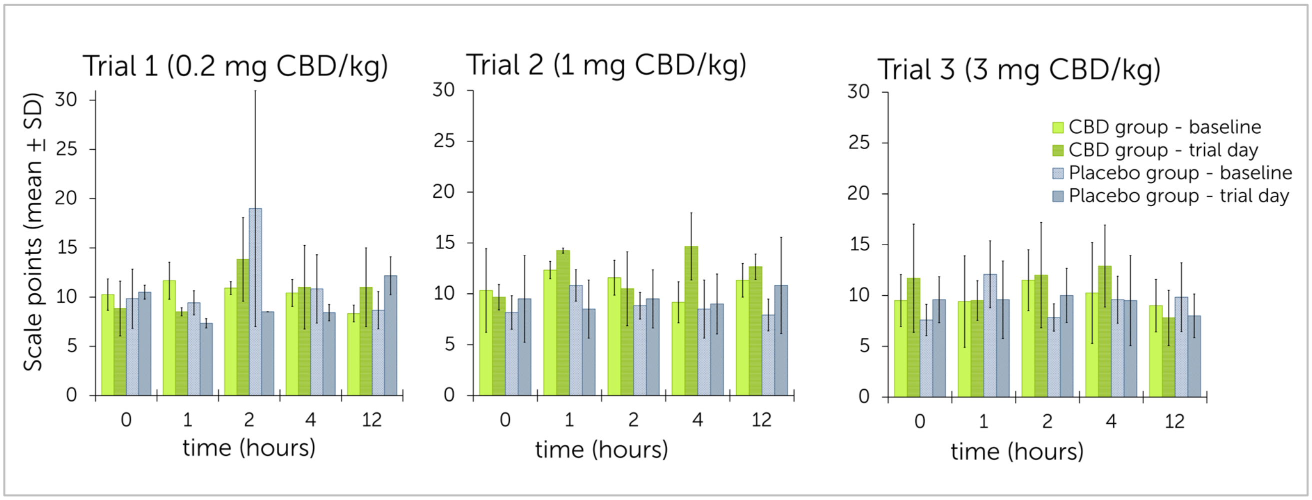

Supplementary Figure 3S: Sedation scores after visual stimulation with a pink cloth following single oral administration of cannabidiol (CBD) paste in escalating doses (0.2 mg CBD/kg; 1 mg CBD/kg; 3 mg CBD/kg) - comparison between values obtained on baseline and trial day for the treatment and control group. Higher scale points relate to a higher level of sedation (Table 1).

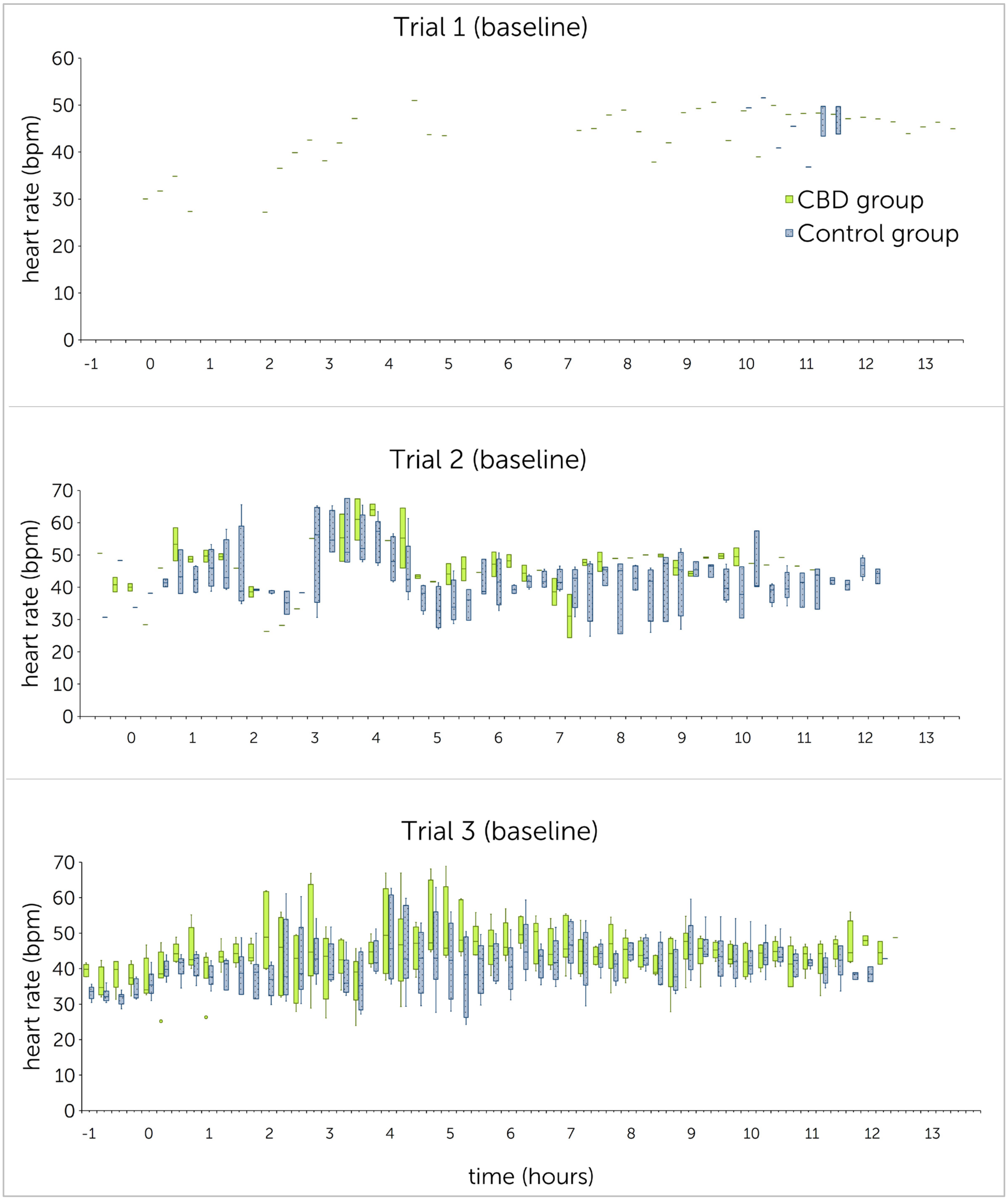

Supplementary Figure 4S: Heart rates (beats per minute (bpm)) prior to single oral administration of cannabidiol (CBD) in three escalating doses (0.2 mg CBD/kg BW; 1 mg CBD/kg BW; 3 mg CBD/kg BW), displayed in 15-minute sections over 12 hours. Due to technical issues, the trial 1 R-R-interval data are partly incomplete.

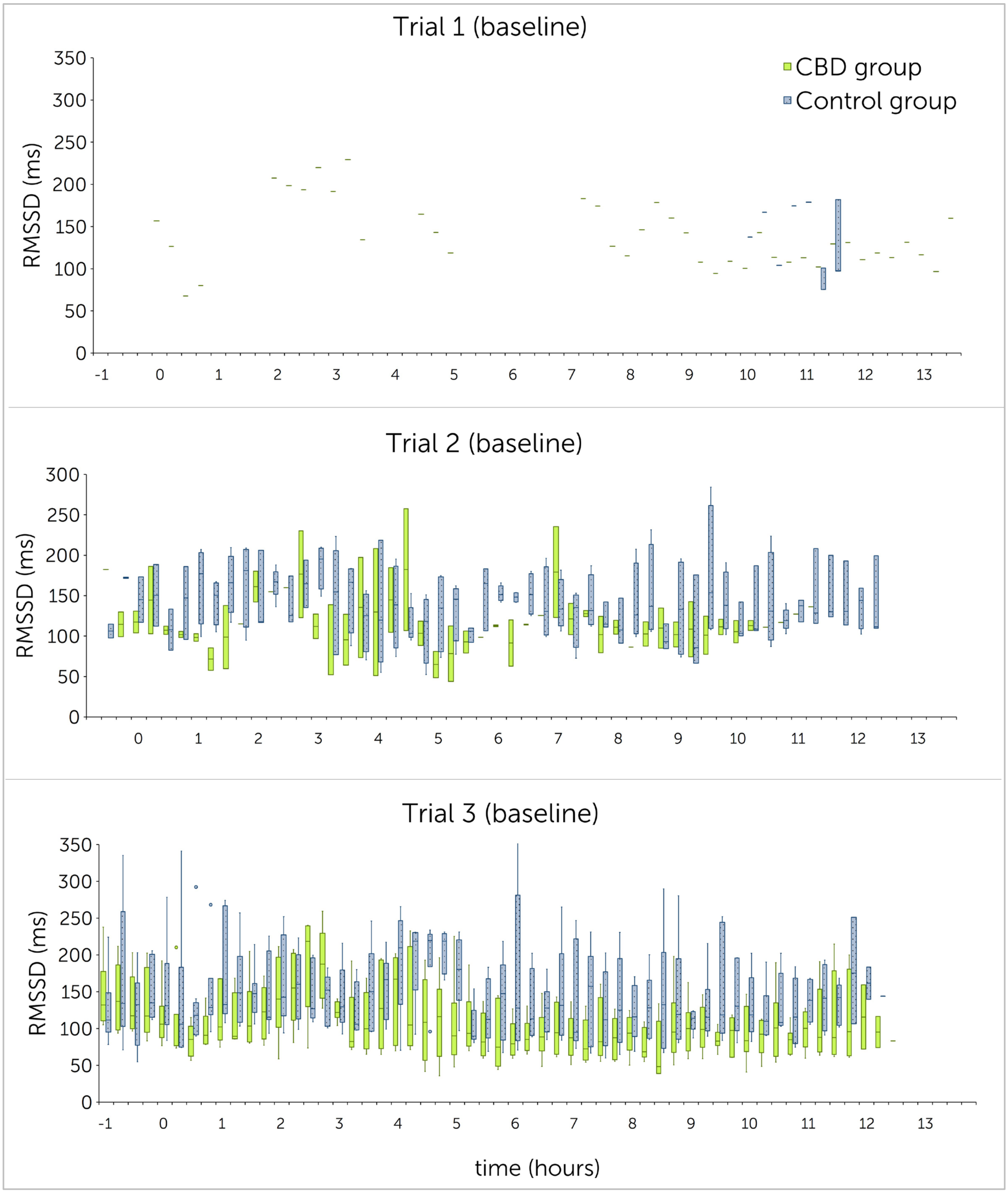

Supplementary Figure 5S: Root mean square of successive R-R interval differences (RMSSD) in milliseconds (ms) prior to single oral administration of cannabidiol (CBD) in three escalating doses (0.2 mg CBD/kg BW; 1 mg CBD/kg BW; 3 mg CBD/kg BW), displayed in 15-minute sections over 12 hours. Due to technical issues, the trial 1 R-R-interval data are partly incomplete.

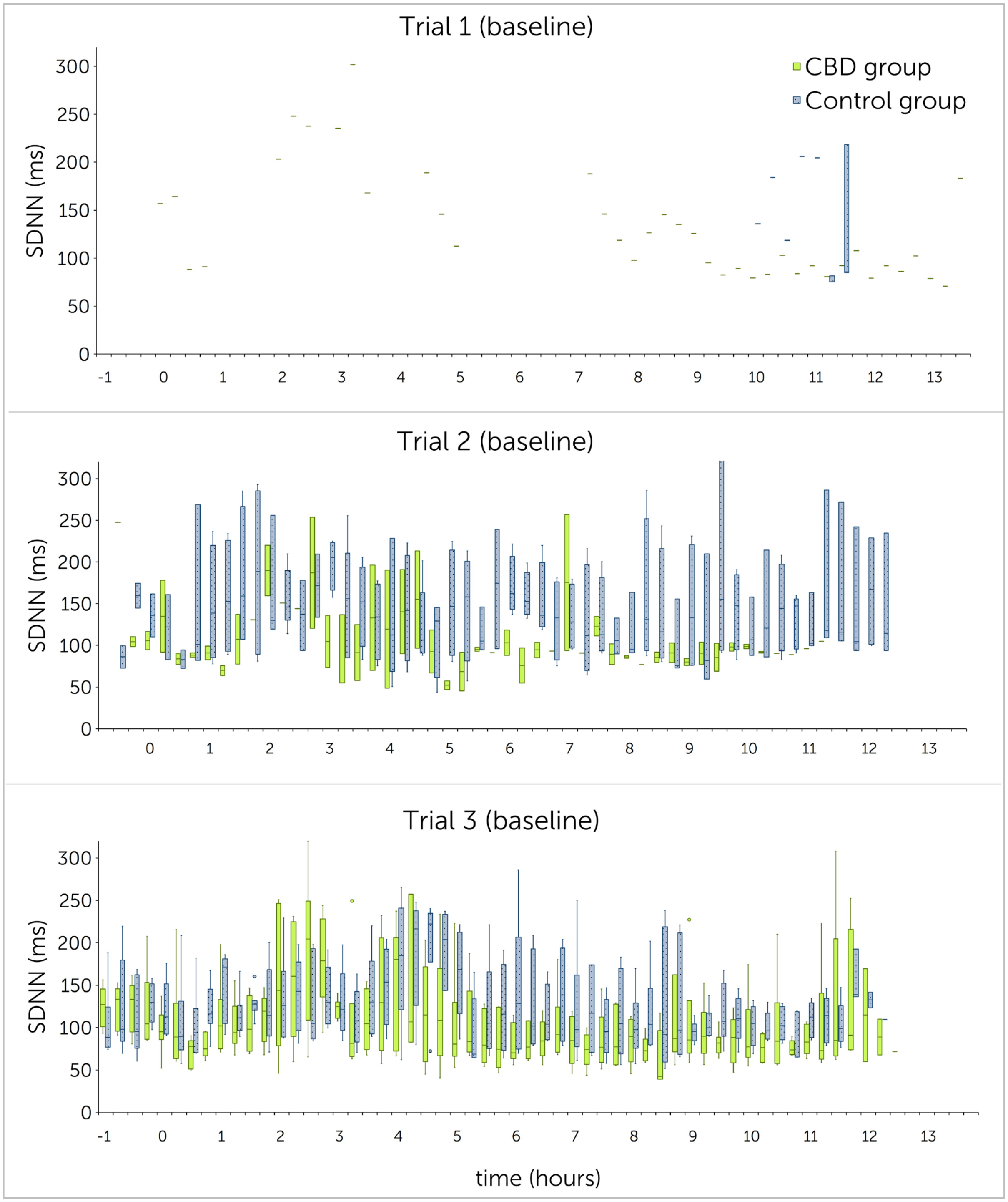

Supplementary Figure 6S: Standard deviations of R-R intervals (SDNN) in milliseconds (ms) prior to single oral administration of cannabidiol (CBD) in three escalating doses (0.2 mg CBD/kg BW; 1 mg CBD/kg BW; 3 mg CBD/kg BW), displayed in 15-minute sections over 12 hours. Due to technical issues, the trial 1 R-R-interval data are partly incomplete.

2 Tables

Supplementary Table 1S: Examples of the facial expression scale (Table 2).

<table>
<tr><td rowspan="6">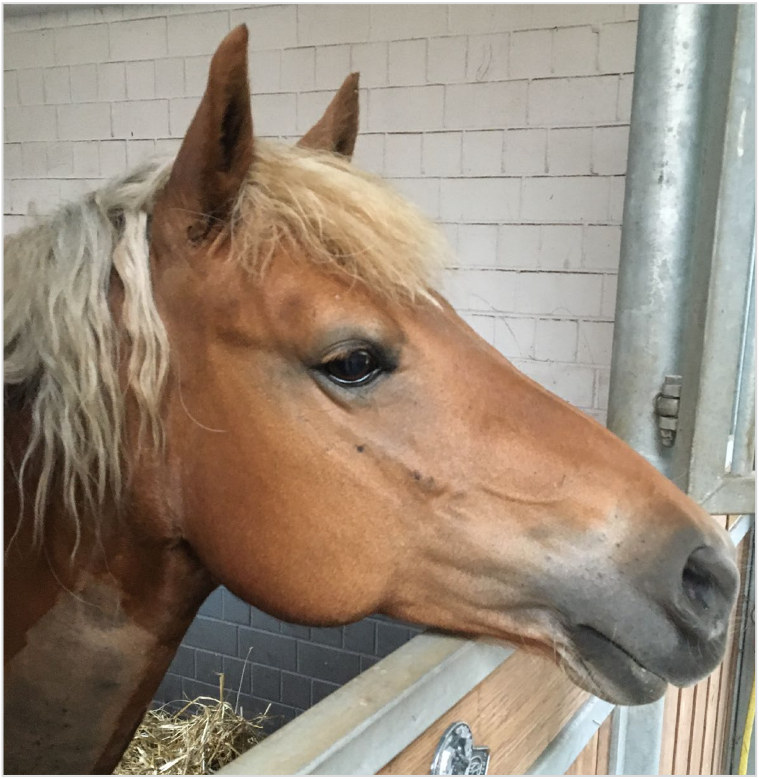</td><td>Orbital opening
2 Eyes completely open</td></tr>
<tr><td>Position of ears
2 Pointed, position of attention</td></tr>
<tr><td>Chewing muscles
2 Moderately present</td></tr>
<tr><td>Lips
2 Loose touching of lips</td></tr>
<tr><td>Nostrils
1 Dilated, outer ring clearly visible</td></tr>
<tr><td>Total sum: 9</td></tr>
<tr><td rowspan="6">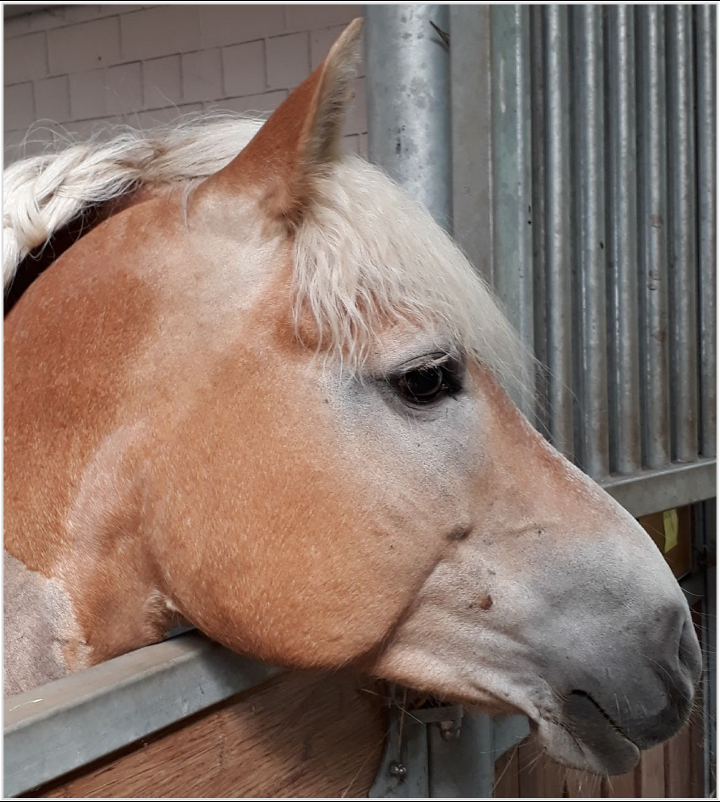</td><td>Orbital opening
2 Eyes completely open</td></tr>
<tr><td>Position of ears
2 Pointed, position of attention</td></tr>
<tr><td>Chewing muscles
2 Moderately present</td></tr>
<tr><td>Lips
2 Loose touching of lips</td></tr>
<tr><td>Nostrils
2 Non-dilated nostrils</td></tr>
<tr><td>Total sum: 10</td></tr>
</table>

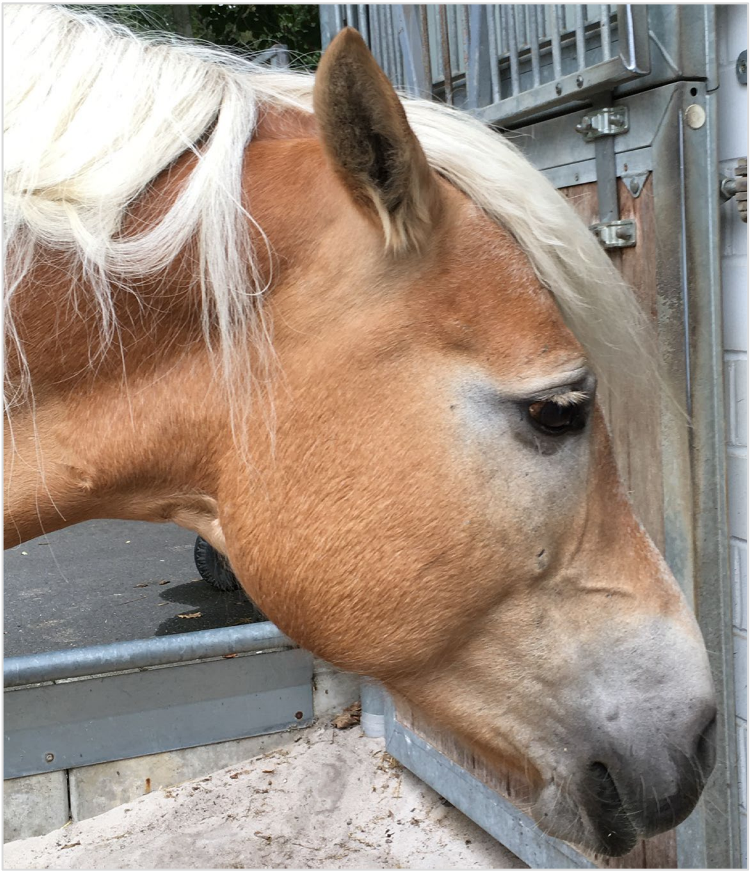	**Orbital opening**<br>3 Eyes partially open (> 50%)<br>**Position of ears**<br>3 Asymmetrical; one ear hanging<br>**Chewing muscles**<br>2 Moderately present<br>**Lips**<br>2 Loose touching of lips<br>**Nostrils**<br>3 Small nostrils, relaxed outer ring<br>**Total sum: 13**
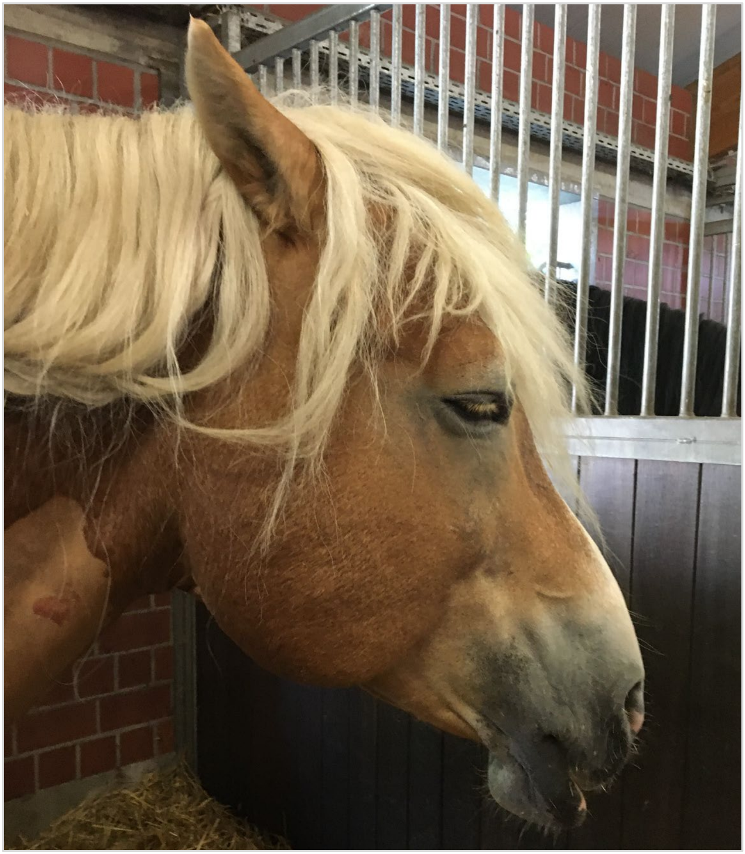	**Orbital opening**<br>4 Eyes almost/completely closed (< 50%)<br>**Position of ears**<br>4 Wide opening between ear tips<br>**Chewing muscles**<br>2 Moderately present<br>**Lips**<br>4 Pronounced relaxation/hanging of one lip<br>**Nostrils**<br>2 Non-dilated nostrils<br>**Total sum: 16**

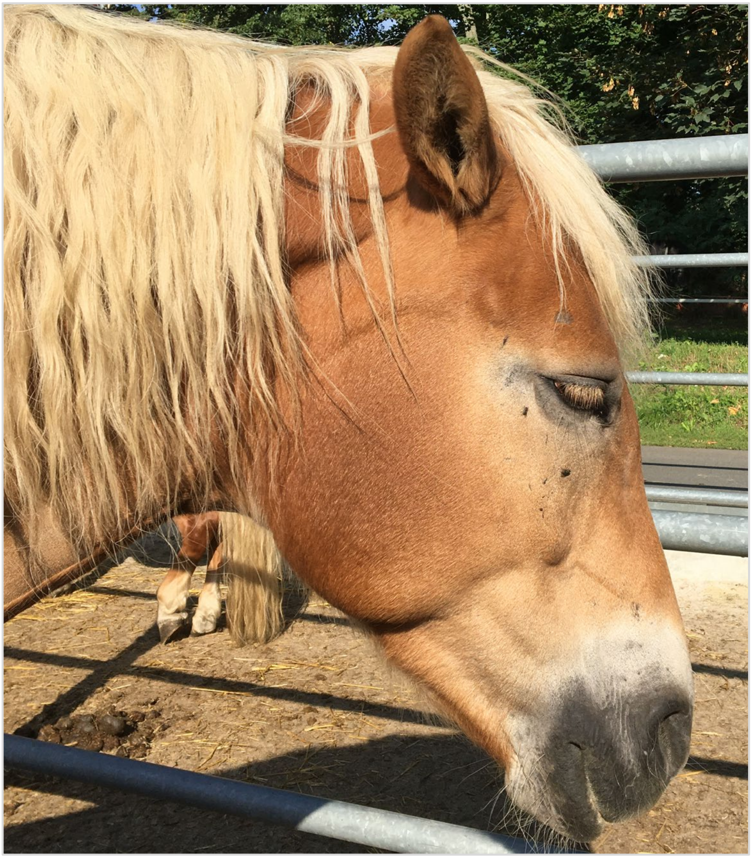	**Orbital opening**<br>4 Eyes almost/completely closed (< 50%)
	**Position of ears**<br>4 Wide opening between ear tips
	**Chewing muscles**<br>3 Not present
	**Lips**<br>3 Slight relaxation of one lip
	**Nostrils**<br>3 Small nostrils, relaxed outer ring
	**Total sum: 17**
